# Electroencephalogram-based adaptive closed-loop brain-computer interface in neurorehabilitation: a review

**DOI:** 10.3389/fncom.2024.1431815

**Published:** 2024-09-20

**Authors:** Wenjie Jin, XinXin Zhu, Lifeng Qian, Cunshu Wu, Fan Yang, Daowei Zhan, Zhaoyin Kang, Kaitao Luo, Dianhuai Meng, Guangxu Xu

**Affiliations:** ^1^Department of Rehabilitation Medicine, Nanjing Medical University, Nanjing, China; ^2^Rehabilitation Medicine Center, Zhejiang Chinese Medical University Affiliated Jiaxing TCM Hospital, Jiaxing, China; ^3^Rehabilitation Medicine Center, The First Affiliated Hospital of Nanjing Medical University, Nanjing, China

**Keywords:** neurorehabilitation, brain-computer interface, BCI, electroencephalography, adaptive, closed-loop

## Abstract

Brain-computer interfaces (BCIs) represent a groundbreaking approach to enabling direct communication for individuals with severe motor impairments, circumventing traditional neural and muscular pathways. Among the diverse array of BCI technologies, electroencephalogram (EEG)-based systems are particularly favored due to their non-invasive nature, user-friendly operation, and cost-effectiveness. Recent advancements have facilitated the development of adaptive bidirectional closed-loop BCIs, which dynamically adjust to users’ brain activity, thereby enhancing responsiveness and efficacy in neurorehabilitation. These systems support real-time modulation and continuous feedback, fostering personalized therapeutic interventions that align with users’ neural and behavioral responses. By incorporating machine learning algorithms, these BCIs optimize user interaction and promote recovery outcomes through mechanisms of activity-dependent neuroplasticity. This paper reviews the current landscape of EEG-based adaptive bidirectional closed-loop BCIs, examining their applications in the recovery of motor and sensory functions, as well as the challenges encountered in practical implementation. The findings underscore the potential of these technologies to significantly enhance patients’ quality of life and social interaction, while also identifying critical areas for future research aimed at improving system adaptability and performance. As advancements in artificial intelligence continue, the evolution of sophisticated BCI systems holds promise for transforming neurorehabilitation and expanding applications across various domains.

## 1 Introduction

Brain-computer interfaces (BCIs) provide a direct communication pathway by bypassing traditional peripheral neural and muscular channels. This innovative capability has increasingly attracted attention across neuroscience, neuroengineering, and clinical rehabilitation fields (Islam and Rastegarnia, 2023). The core objective of BCI technology is to facilitate non-muscular communication, thus enabling individuals with severe motor impairments to interact with their environment (Islam and Rastegarnia, 2023). Among the diverse BCI technologies, electroencephalogram (EEG)-based BCIs are particularly favored due to their non-invasive nature, user-friendly operation, and cost-effectiveness. Recent advancements have seen EEG-based BCI systems incorporate adaptive closed-loop control capabilities. These systems dynamically adjust their parameters based on the user’s brain activity, thus enhancing the system’s responsiveness and efficacy (Shupe et al., 2021). Adaptive closed-loop BCIs offer real-time modulation, bidirectional communication, and closed-loop feedback, which are especially advantageous in neurorehabilitation. They provide personalized therapeutic interventions by continuously monitoring and adjusting to the user’s neural and behavioral responses (Dangi et al., 2013). The adaptive mechanism inherent in these BCIs allows for real-time adjustments in response to fluctuations in EEG signals. Machine learning algorithms typically drive these adjustments, constantly refining the BCI’s decoding parameters to optimize user-system interaction (Arvaneh et al., 2017). For instance, as a user’s neural responses evolve during rehabilitation, the BCI can modify feedback parameters to ensure that therapeutic stimuli remain effective and tailored to individual needs (Soekadar et al., 2015). Bidirectional communication is a crucial feature of these systems, encompassing both the decoding of neural signals to control external devices and the provision of feedback to the user. This feedback loop is essential for neurorehabilitation, enabling users to perceive the real-time impact of their neural activity, thus reinforcing learning and fostering neuroplasticity (Bronte-Stewart et al., 2020). Feedback is delivered through various sensory modalities, such as visual, auditory, or haptic signals, facilitating a dynamic and reciprocal interaction between the brain and external devices (Leeb et al., 2007). Closed-loop feedback systems continuously monitor EEG signals and make instantaneous adjustments to system outputs, thereby allowing BCIs to adapt effectively to changes in the user’s neural state (Lotte et al., 2018). This is in stark contrast to open-loop systems, which lack real-time feedback and cannot accommodate ongoing changes in neural conditions (Zhang et al., 2021). In neurorehabilitation, the real-time feedback of closed-loop BCIs aligns system responses with the user’s evolving cognitive and motor states, significantly enhancing rehabilitation outcomes (Ramos-Murguialday et al., 2019). In summary, adaptive closed-loop BCIs present notable advantages in neurorehabilitation. They support the personalization of rehabilitation protocols to meet individual patient needs, potentially accelerating recovery and improving functional outcomes through real-time neural feedback. Additionally, the bidirectional nature of these systems fosters active engagement and learning, crucial components for effective rehabilitation (Lebedev and Nicolelis, 2017). This progress underscores the potential for substantial advancements in neurorehabilitation technologies and offers innovative approaches for addressing neurological disorders.

## 2 Dynamic neural mechanisms in adaptive closed-loop BCI

BCIs are a transformative technology that harnesses brain signals to control external devices or deliver feedback to the brain, fundamentally changing how the central nervous system (CNS) interacts with the environment (Abiri et al., 2019). A BCI system is composed of three critical components: signal acquisition and processing, decoding and control, and feedback mechanisms (Fetz, 2015). The signal acquisition module captures EEG signals from scalp electrodes, which are then preprocessed through filtering, noise reduction, and feature extraction to improve signal quality and detectability. The decoding module interprets these features to discern the user’s motor intentions or motor imagery (MI), converting them into commands that govern external devices. Meanwhile, the feedback module provides sensory feedback, such as electrical stimulation, tactile sensations, or virtual reality cues, to inform the user of the outcomes of their actions (Nojima et al., 2022). This bidirectional flow of information—from decoding neural signals to delivering feedback—is pivotal for the BCI’s adaptive capabilities, allowing the system to adjust its operations in real-time based on changes in the user’s neural activity. This enhances the user’s understanding of and interaction with the system, thereby improving overall system performance (Wolpaw et al., 2020). Closed-loop adaptive BCI systems, especially those based on EEG, further deepen this interaction by integrating multiple brain signals from various CNS regions, with a focus on cortical and subcortical structures (Wang et al., 2019). The defining characteristic of these systems is their capacity to enable dynamic interactions between the user and the BCI. The system decodes neural signals generated by the user, transforming them into commands for external devices—this represents the initial communication pathway, from user to system (Wolpaw et al., 2020). Simultaneously, the system provides real-time feedback based on the decoded signals and system output, delivered through visual, auditory, or tactile stimuli (Deo et al., 2021). This feedback loop is crucial as it informs the user about the effectiveness of their neural activity in controlling the system, establishing the second communication pathway, from system to user (Wolpaw et al., 2002). This continuous feedback mechanism allows for ongoing adaptation, where users can refine their mental strategies while the system concurrently adjusts its parameters in response to neural feedback (Lebedev and Nicolelis, 2006; Khorev et al., 2024). This dynamic, bidirectional interaction is vital for optimizing neurorehabilitation outcomes, as it facilitates continuous adjustments in both user engagement and system responsiveness, ultimately enhancing the effectiveness of personalized and adaptive therapeutic interventions tailored to the specific needs of each patient (Daly and Wolpaw, 2008).

## 3 Major category

### 3.1 BCI based on event-related potentials

Event-related potentials (ERPs) are crucial signals in BCI systems, reflecting neural information processing in response to stimuli (Sur and Sinha, 2009; Yu et al., 2016). These signals capture time-locked brain electrical activities associated with both physical and mental tasks, functioning as a non-invasive cognitive imaging method known for its high temporal resolution, as detected in EEG recordings (Ma et al., 2021). In the context of BCI applications, ERPs serve various purposes, including the recognition of user intent and emotions, as well as the examination of cognitive functions like attention, memory, language, and emotional processing (Xu et al., 2020). ERPs encompass both sensory responses, such as visual evoked potentials (VEPs), and more complex components, including N2 and P3, which are modulated by higher-order cognitive processes. VEPs, elicited by visual stimuli, are most prominently detected in the occipital region and are indicative of visual perception, transmission, and processing. Subtypes of VEPs include transient visual evoked potentials (TVEP), steady-state visual evoked potentials (SSVEP), motor visual evoked potentials (mVEP), and code-modulated visual evoked potentials (cVEP) (Shirzhiyan et al., 2020). While VEPs are primarily associated with sensory processing, components such as N2 and P3 are linked to cognitive functions. The N2 component is typically involved in conflict monitoring and cognitive control, originating mainly in the anterior cingulate cortex (Folstein and Van Petten, 2008). The P3 component, characterized by a positive deflection approximately 300–400 ms after stimulus presentation, is associated with stimulus evaluation, attention allocation, and decision-making processes, primarily engaging the parietal and frontal regions (Xu et al., 2013). Given their involvement in cognitive processing, N2 and P3 components should be distinguished from purely sensory ERPs, as they reflect complex interactions within the brain’s cognitive networks. In BCI systems, the P3 component is particularly valuable due to its strong correlation with cognitive states, offering a high signal-to-noise ratio and consistent temporal characteristics (Picton, 1992; Polich, 2007). The information encoded in modulated ERP signals, including those related to N2 and P3, can be decoded using advanced signal processing and classification techniques. Compared to spontaneous EEG rhythms that do not require external cues, ERP signals like P3 provide significant advantages, including reduced user variability and stable spatiotemporal features, making them highly suitable for BCI applications (Pan et al., 2022). Recent advancements in signal modulation and demodulation technologies have further enhanced the efficiency of ERP-based BCI systems, leading to higher communication speeds (Craik et al., 2019; Savić et al., 2023).

### 3.2 BCI based on sensorimotor rhythms

Sensorimotor rhythms (SMRs) represent brainwave activity that falls within the frequency range of 8–30 Hz and reflects the rhythmic electrical patterns seen in groups of neurons. These brainwaves are closely tied to our ability to maintain control over rhythmic movements and basic behaviors (Wang et al., 2019; Wolpaw and Thompson, 2023). Research into the field of neurophysiology has shown that alpha waves (8–12 Hz) and beta waves (13–30 Hz) can be influenced by actual movement and motor planning. When tasks are being performed, SMRs tend to show a decrease in amplitude or power in the lower frequency spectrum, leading to what is known as event-related desynchronization (ERD). On the other hand, an increase in amplitude within a specific frequency range is referred to as event-related synchronization (ERS) (McFarland et al., 2006). By observing the distinct changes in SMRs through EEG measurements, it becomes possible to classify the brain’s activity during the planning or visualization of various limb movements. This forms the basis for SMR-based Brain Computer Interfaces (Pfurtscheller et al., 2006). In brain-computer interfaces based on SMRs, the primary method for controlling SMRs is through MI. Research indicates that individuals can be trained to control the amplitude of SMRs through MI, which can then be used for operating a cursor and spelling in BCIs. Through the use of spatial filters and classifiers to teach participants how to produce distinct MI states, BCI’s coadaptive learning system facilitates simultaneous learning by both the brain and the machine. This system is capable of recognizing more complex spatiotemporal patterns of ERD/ERS when imagining movements of various body parts, such as the hands, feet, and tongue (Fumanal-Idocin et al., 2022). Currently, there is a growing body of research exploring the potential uses of SMR-based BCIs in motor rehabilitation, cognitive improvement, and other areas.

### 3.3 Hybrid BCI

Hybrid BCI systems blend BCI with additional physiological or technical signals to combine multiple input streams, with the goal of enhancing the accuracy and/or speed of communication of traditional BCIs and broadening their user base (Zhu et al., 2020). Depending on the mode of the secondary signal, hybrid BCIs can be categorized as either simple or complex. Simple hybrid BCIs commonly incorporate two EEG modalities to enhance BCI performance. Widely utilized simple hybrid EEG modalities currently include combinations of ERP and SMR, like SSVEP-MI and P300-MI, as well as pairs of ERP signals such as P300-SSVEP and N2pc-SSVEP (Barios et al., 2019). Apart from hybrid EEG signals, other brain signals can be used to create simple hybrid BCIs, for instance, an EEG-BCI utilizing functional near-infrared spectroscopy technology (Liu et al., 2022). On the other hand, complex hybrid BCIs combine EEG with various non-neuronal control signals to achieve more stable and reliable control. These control signals involve physiological signals such as electromyography, electrooculography, heart rate, or signals from other existing input devices like eye-tracking systems, often originating from residual muscle function (Jiang et al., 2014; Nann et al., 2021).

Hybrid BCIs can be further categorized into synchronous and sequential types based on how they process multiple input signals over time. Synchronous hybrid BCIs combine information from various input signals, requiring the integration of multiple inputs in processes such as extracting features, combining features, and making classification decisions. These signals all have the same intentional control goals to improve accuracy. Research has shown that BCI systems that combine SSVEP and P300 signals can greatly improve the recognition of target fixation, leading to higher accuracy in selecting targets and faster data transfer rates (Tang et al., 2022). On the other hand, sequential hybrid BCIs involve the consecutive operation of two systems, with one usually functioning as a switch or selector, and the other as a conventional BCI controller (Ma et al., 2017).

## 4 Applications in neurorehabilitation

The primary purpose of EEG-based adaptive closed-loop BCI in rehabilitation is to substitute and recover damaged neural functions. These BCIs assist individuals in regaining authority over various environments and tasks, such as tasks involving computers (typing documents, browsing the internet) (He et al., 2020), controlling the environment (adjusting lights, temperature, TV) (Daly and Wolpaw, 2008), and using mobile devices (controlling electric wheelchairs, neuroprosthetics, orthoses) (Li et al., 2013; Kotov et al., 2019). Additionally, they can be incorporated into rehabilitation treatments. By promoting activity-dependent neuroplasticity, BCIs aid in the reinstatement of normal CNS operation (Halme and Parkkonen, 2022).

### 4.1 Promote recovery of motor function

Currently, EEG-based adaptive closed-loop BCI systems have been combined with a variety of tools, such as FES on the surface and inside the body, neuroprosthetics, exoskeletons, orthoses, virtual limbs, and robotic arms. This merging introduces innovative ways to carry out essential tasks for patients who are unable to move (Rupp, 2014; Tidoni et al., 2017). By joining BCI systems with these treatments, patients can engage in repetitive training for upper limb movements at a high frequency, leading to increased involvement in their recovery process (Remsik et al., 2016). This method is beneficial because it creates opportunities for treatment in individuals with limited motor function, enabling them to regain control and reestablish connections between the brain and nerves in the body (Kim et al., 2022). In a study by Kreilinger and colleagues, it was shown that patients with spinal cord injuries undergoing neuroprosthesis training using FES in combination with MI-BCI were able to use EEG-controlled shoulder sensors to select between grasping, elbow flexion, or resting functions (Kreilinger et al., 2013). Another research demonstrated that the efficacy of non-invasive FES-BCI hand rehabilitation in enhancing neurological function recovery in individuals with subacute cervical spinal cord injury exceeded that of FES alone (Osuagwu et al., 2016). Kosmyna et al. combined BCI with biofeedback and observed that biofeedback had the ability to shift participants’ focus toward the current task, resulting in improved performance on comprehension assessments. Through a study involving non-invasive BCI technology for robotic arm control, it was discovered that repetitive training could induce EEG-triggered sequences, ultimately enhancing the control performance of the robotic arm (Kosmyna and Maes, 2019). Presently, research into using BCI systems for the control of lower limb movements following spinal cord injury is scarce (Athanasiou et al., 2017). In recent years, there has been a growing interest in the intersection of BCI and wearable exoskeletons, hinting at potential for gait training in diverse neurological conditions in the coming years (Murphy et al., 2017). Studies have shown that the motor intentions of patients, as well as their MI, are crucial in promoting cortical reorganization and aiding in the recovery of motor functions in paralyzed limbs (Langhorne et al., 2009; Maier et al., 2019). Non-invasive neuroregulation techniques such as EEG-based adaptive closed-loop BCI systems have been developed to enhance patients’ perception and evaluation of their motor status through various methods like electrical stimulation, visual and tactile feedback, and virtual reality training. These systems are effective in promoting cortical reorganization, modulating neuroplasticity, and improving motor function (Gao et al., 2021). A recent study conducted by Sinha et al. involved 23 stroke patients with upper limb motor disorders who underwent EEG-based FES-BCI interventions. The results showed a significant increase in interhemispheric resting-state functional connectivity within the motor network, as well as notable improvements in upper limb motor function tests and stroke impact scale outcomes (Sinha et al., 2021). Research on ankle dorsiflexion MI-BCI interventions in both healthy individuals and stroke patients revealed that MI-BCI training had a positive impact on the excitability of the lower limb motor cortex in stroke patients, leading to improvements in lower limb motor recovery (Chung et al., 2015). Furthermore, two separate studies highlighted the feasibility and stability of EEG-based SSVEP-BCI systems in enhancing the recovery of upper limb motor function in patients with post-stroke motor impairments (Chi et al., 2022; Guo et al., 2022). EEG-based adaptive closed-loop for BCI can function as a non-invasive technology for neural control by detecting the motor intentions or MI of patients and translating them into corresponding control instructions. These instructions prompt external devices to carry out the appropriate limb movements, presenting a more natural and versatile means of interaction and boosting patient confidence and satisfaction in rehabilitation (Bai et al., 2020). Through EEG signal-based identification of patients’ motor intentions or MI, Bundy et al. implemented a 12-week exoskeleton training regimen for 10 stroke recovery patients with moderate to severe impairments in upper limb motor function. The data illustrated an average improvement of 6.2 points in scores on upper limb motor function assessments with the aid of BCI, which displayed a notable relationship with enhanced BCI control proficiency (Bundy et al., 2017). In a study by Cantillo-Negrete et al., the viability of EEG-BCI in conjunction with a robotic arm was examined in patients with functional impairments in the upper limb post-stroke, with an assessment of the integrity of the cortical spinal tract in the affected hemisphere using transcranial magnetic stimulation. The outcomes suggested that the combined utilization of EEG-driven BCI and a robotic arm could trigger mechanisms associated with neuroplasticity (Cantillo-Negrete et al., 2021). Moreover, a BCI-controlled robot-assisted therapy system was proposed by researchers, demonstrating promising efficacy in advancing recovery of lower limb function among stroke patients (Johnson et al., 2018). Moreover, adaptive closed-loop BCI systems utilizing EEG serve as valuable tools for neural monitoring without the need for invasive procedures. These systems are able to analyze brain signals in real-time, assessing movement status and individual requirements. They automatically adjust feedback modes, intensity, control strategies, difficulty levels, monitoring indicators, and standards based on unique variations and training progress, resulting in more effective and personalized limb movement rehabilitation exercises (Laiwalla and Nurmikko, 2019). A study conducted by Mansour et al. found that alpha rhythms are linked to motor learning, while beta rhythms are involved in facilitating communication between the motor cortex and the paralyzed upper limb in BCI-assisted robot interventions, indicating a compensatory function of the cortical layer in stroke patients with severe motor deficiencies (Mansour et al., 2022). Research by Ang et al. demonstrated that tDCS May potentially enhance motor imagery in stroke patients when combined with robot feedback in the context of MI-BCI applications (Ang et al., 2015). Additionally, Rea et al.’s study showed that utilizing functional near-infrared spectroscopy technology in combination with BCI for gait training could provide new insights on correcting gait abnormalities in stroke patients (Rea et al., 2014). The integration of BCI technology with these therapeutic methodologies offers valuable guidance for treating movement disorders resulting from neurological conditions.

### 4.2 Promote recovery of sensory function

Skin sensory input plays a vital role in motor control, as it is necessary for flexible grasping and manipulation of objects. Sensations serve as important indicators during object interaction, providing key information on slip or contact force to enhance human-machine interactions (Johansson and Flanagan, 2009). Adaptive colsed-loop BCI systems, which interpret brainwave signals, achieve bidirectional information transmission, facilitating sensory function recovery for patients. Lower limb amputees face challenges in activities like stair climbing and walking on uneven terrain due to decreased sensory feedback, whereas BCIs using EEG data show high accuracy in recognizing lower limb movements (Dillen et al., 2022). Integrating BCI with intelligent control algorithms allows for adaptive adjustments based on individual differences and rehabilitation progress, improving rehabilitation efficiency and accuracy. A major goal is to develop responsive prostheses that can transmit essential sensory information to the brain. Stimulating the central nervous system for sensory signal input involves transmission from the cerebral cortex along the spinal cord, or direct stimulation of the dorsal root ganglia or rootlets. Dorsal root ganglia have distinct advantages as they activate sensory pathways independently from motor efferents, avoiding interference with electromyographic control. Additionally, different minimally invasive methods make it easier to reach the dorsal ganglia roots. Furthermore, a study conducted by Cui and colleagues highlights the distinct ability of BCI rehabilitation systems to offer two-way stimulation at the site of spinal cord injury, leading to favorable results for individuals with total spinal cord injuries (Cui et al., 2023). The intensity of sensations can be categorized according to the strength of the stimulus, and comparable results have been obtained through surface-based stimulation or EEG, both of which can provide sensory feedback for specific tasks or enhance bodily awareness in prosthetics. Relevant studies have demonstrated that intracortical microstimulation in the primary somatosensory cortex region 1 of individuals with limb paralysis can elicit natural feelings (Flesher et al., 2016). Over time, these feelings stabilize and can be directed to specific fingers, with the intensity being adaptable. Additionally, Collins and colleagues noted that the feeling of owning prosthetic devices can be induced by simultaneously stimulating the hand region of the somatosensory cortex and experiencing tactile sensations from interacting with a rubber hand (Collins et al., 2017). It is important to mention that when the stimulation targets somatosensory cortex regions not associated with the hand, it does not create illusions, emphasizing that multisensory integration follows basic spatial and temporal guidelines. These research findings emphasize the brain’s ability to merge “natural” visual input with direct cortical-somatosensory stimulation, creating a multisensory perception that attributes the prosthetics to the individual’s own body. These results indicate that BCIs can trigger a sense of ownership over multisensory and prosthetic body parts, bypassing the peripheral nervous system, which is essential for patients with spinal or nerve injuries lacking peripheral sensory feedback.

## 5 Challenges in practical application development

### 5.1 Classifier design and signal processing

#### 5.1.1 Challenges of online and offline classifiers

While most classification methods have been validated in offline settings, practical BCI systems require real-time online operation (Abiri et al., 2019). Consequently, classifiers must demonstrate high computational efficiency, rapid calibration capabilities, and robust resistance to EEG signal noise in real-world environments. Online evaluation should be the standard practice in classifier development rather than an exception. Although many classification methods have been assessed exclusively under offline conditions, the primary utility of BCI applications lies in their online, real-time operation (Lotte et al., 2018). Therefore, it is imperative that these classifiers be studied and validated in online settings to ensure they meet the demands of computational efficiency, ease of rapid calibration, and robustness against EEG noise in practical environments (Perdikis et al., 2016). Indeed, the routine online evaluation of classifiers is essential, as classifiers that cannot function effectively in real-time environments hold limited research value.

#### 5.1.2 Complexity of signal processing and decoding

Despite extensive research on decoding methods and signal processing algorithms, extracting useful information from EEG signals remains challenging due to the inherently low signal-to-noise ratio (SNR), particularly in the context of controlling multi-degree-of-freedom neuroprosthetic systems (He and Wu, 2020). This challenge underscores the need for the development of more robust, accurate, and fast online algorithms. While current approaches predominantly rely on static classifiers, adaptive classifiers and decoders offer the potential to enhance performance by compensating for the nonstationary characteristics of EEG signals. Recent studies have explored advanced techniques, such as EEG source localization and active data selection (Yger et al., 2017), which May further improve classification performance. Additionally, advanced machine learning and deep learning methods hold promise for extracting additional features, thereby increasing classification accuracy (Yin et al., 2017; Wang et al., 2023). Addressing the nonstationarity of EEG signals through the use of adaptive classifiers and decoders represents a significant advancement (Zanini et al., 2018). Moreover, standardized systems are crucial for evaluating the performance of decoding algorithms across specific applications and BCI systems (Zeyl et al., 2016).

#### 5.1.3 Challenges of transfer learning and domain adaptation

Transfer learning and domain adaptation techniques are regarded as critical for achieving zero calibration in BCI research. However, these technologies require substantial development and optimization before they can become standard tools. Current research primarily focuses on scenarios where tasks in the source and target domains are identical, particularly in motor imagery tasks, facilitated by the availability of BCI competition datasets (Lotte and Guan, 2011; Arvaneh et al., 2013; Wu et al., 2022). Additionally, other paradigms, such as P300 spellers and visual/spatial attention tasks, have also attracted research interest. Nevertheless, variability in data between subjects or sessions remains a significant challenge for transfer learning and domain adaptation techniques (Kindermans et al., 2014). To address these challenges, adaptive classification methods have been integrated into the field of transfer learning. For example, researchers have enhanced classification performance by learning dictionaries of spatial filters and adapting them to the resting-state EEG characteristics of target subjects (Morioka et al., 2015). Another approach involves transferring features from target domain data to the source domain, thereby enabling classifiers trained on source domain data to be applied effectively to target data. Recently, the application of optimal transport methods in domain adaptation has gained traction, particularly in the session-to-session transfer of P300 data, which facilitates the effective transformation of probability distributions across different domains (Courty et al., 2017). Transfer learning is crucial for improving decoding performance across sessions and subjects, thereby reducing the calibration demands of BCI systems and enhancing their usability and user acceptance (Shao et al., 2015). Although transfer learning methods are not yet perfected, their robustness, combined with adaptive classifiers, represents a cutting-edge direction in BCI research, and is pivotal in realizing zero-calibration operational modes.

#### 5.1.4 Limitations of information transfer rate

The ITR of current BCI systems is relatively low, constraining their application range and efficiency. ITR is a key metric for evaluating BCI performance, and its enhancement depends on various factors, including the number of targets, classification accuracy, and target selection duration (Zhang and Guan, 2010). In motor imagery tasks, precise classification of movements involving the hands, feet, and tongue is essential, with multi-dimensional continuous control needed to expand the target range. To reduce the number of flashes required for character recognition, a P300-BCI system utilizing a 12 × 7 matrix with unique flashing character patterns has been proposed (Jin et al., 2011). Furthermore, traditional multiplexing methods, such as time-division multiplexing (TDMA), frequency-division multiplexing (FDMA), code-division multiplexing (CDMA), and spatial-division multiplexing, have been successfully applied to BCI systems, effectively reducing target selection time (Chen et al., 2014). Improving classification accuracy is crucial for increasing ITR, which can be achieved through two main approaches: first, by implementing advanced signal processing algorithms, such as artifact removal, spatial filtering, and SNR enhancement, to strengthen task-related EEG signals; and second, by leveraging machine learning techniques for feature selection, combination, and classification (Bin et al., 2011). Enhancing SNR directly influences target detection accuracy, thereby improving the ITR of BCI systems. To further elevate ITR, researchers can explore increasing category diversity and developing more complex BCI applications. Additionally, adaptive methods such as dynamic stopping and machine learning techniques, including single-trial classification, can effectively reduce target identification time, thereby further enhancing the ITR of BCI systems (Schreuder et al., 2013).

### 5.2 User experience

#### 5.2.1 User compliance and fatigue issues

The prolonged training requirements of BCI systems can lead to user fatigue and reduce compliance (Holz et al., 2015). Due to individual differences and session-to-session variability, BCI systems often require calibration data before each use. This calibration process is time-consuming and cumbersome, further exacerbating user fatigue (Sellers et al., 2010). Although zero-training or universal BCI models have been proposed in recent years to reduce the reliance on individual calibration, these methods are still under development and not yet fully mature. Additionally, the user’s subjective experience is a critical factor influencing training outcomes; a lack of interest and engagement May undermine the effectiveness of the training. Research indicates that EEG-based BCI systems, particularly those integrating motor imagery, play a significant role in clinical rehabilitation (Padfield et al., 2019). To shorten training time, some studies have explored predictive models based on resting-state EEG and dynamically adjusted interaction modes between the user and the machine, leading to co-adaptive learning models (Blankertz et al., 2010). These approaches show potential for reducing training time and enhancing user experience, but they require further validation and optimization.

#### 5.2.2 Challenges in closed-loop control systems

Closed-loop BCI systems aim to achieve co-adaptation between the user and the machine, optimizing system control performance through mutual learning and adjustment (Meng et al., 2016). In an ideal closed-loop BCI system, the user should act as a minimally involved guide, providing high-level supervisory control, while the BCI system, as an intelligent agent, handles lower-level control tasks (Li et al., 2014). However, current EEG-based BCI systems predominantly rely on visual feedback, and other forms of artificial sensory feedback require further exploration to enhance the perception capabilities of closed-loop control systems. Closed-loop control systems are often described as “dual-learner systems,” where both the user and the computer participate in executing control tasks (Iturrate et al., 2013). This system employs shared and hybrid control mechanisms, with the user generating high-level commands and the traditional control system managing low-level tasks (McMullen et al., 2014). The ideal closed-loop BCI system would position the user as a supervisor, overseeing the autonomous external system through cognitive monitoring rather than directly participating in every control command (Cunningham et al., 2011). To achieve this advanced level of closed-loop control, researchers are exploring various feedback mechanisms, such as brain stimulation, haptic feedback, and somatosensory stimulation, to enhance control accuracy and user experience (Broccard et al., 2014; Yuan and He, 2014).

### 5.3 Device technology

Although EEG technology has been extensively researched due to its advantages of portability, low cost, and high temporal resolution, its practical applications continue to encounter significant challenges. Notably, issues such as user discomfort and vulnerability to artifacts persist, particularly in dry electrode technology, where signal quality deteriorates with prolonged use. This deterioration poses a substantial barrier to the widespread application and commercialization of BCI technology (Mridha et al., 2021). To address these limitations, advancements in EEG technology are being actively pursued, with a specific emphasis on the development of mobile BCI systems. These systems have attracted considerable attention within the research community. Alternative approaches, such as the use of ear electrodes or intra-aural electrodes, have been proposed to enhance the accuracy of signal acquisition (Wang et al., 2017). Furthermore, the integration of advanced classification algorithms, including support vector machines, neural networks, and decision trees, has markedly improved the effectiveness of feature extraction (Gao et al., 2023). In parallel, the ubiquity of mobile devices like smartphones has created new opportunities for the deployment of mobile BCI systems. For instance, Sun et al. introduced a smartphone-based brain-computer interface home care system (HCS) that utilizes a single electrode to capture EEG signals, providing daily support for patients with motor impairments and demonstrating significant usability potential (Sun et al., 2020). Similarly, another smartphone-driven BCI system shows promise as an effective tool for restoring communication capabilities in patients with severe motor impairments by establishing robust communication and control links between brain signals and external devices (Velasco-Álvarez et al., 2021).

### 5.4 Standardization

The issue of standardization is particularly pressing in the field of EEG-based BCIs, especially regarding data analysis and performance evaluation (Dal Seno et al., 2010). Currently, there is a significant lack of unified standards for EEG data analysis. This absence of standardization hinders the comparison of research findings, as different studies May utilize varying data processing workflows and analytical methods. Such inconsistency not only impedes researchers’ ability to interpret existing results but also creates uncertainty for new investigators in choosing suitable analytical approaches, potentially influencing the trajectory of their research (Silvoni et al., 2011). Moreover, the lack of standardized data analysis methods obstructs the fair comparison of research outcomes, complicating the synthesis and evaluation of results across studies (Lebedev and Nicolelis, 2006). Therefore, the establishment of a recognized and standardized framework for BCI evaluation metrics is critically important. A unified evaluation system would provide clear guidelines, ensuring the comparability of different research outcomes and thereby facilitating overall progress in the BCI field. In addition, the inconsistency in performance evaluation standards poses another significant challenge. Different studies often adopt performance metrics tailored to their specific research needs, making cross-study comparisons difficult (Pfurtscheller and Neuper, 1997). For instance, while some studies May prioritize classification accuracy, others might focus on system response time or user experience (Ramos-Argüelles et al., 2009). This diversity in evaluation criteria complicates the comparative analysis of research findings, thereby slowing technological advancement and innovation within the field. Consequently, the development of standardized BCI evaluation criteria would not only help to align research efforts but also promote the overall advancement of the field by enabling effective comparison and synthesis of research outcomes. A standardized evaluation framework would allow researchers to assess technologies against a common benchmark, thereby improving the transparency and reproducibility of research findings.

## 6 Conclusion

In summary, EEG-based adaptive closed-loop BCI systems present significant potential for advancing neural rehabilitation, offering crucial opportunities to enhance the quality of life and social interactions of patients with motor impairments. However, several challenges must be addressed to maximize their effectiveness. First, improvements in the control and design of BCI systems are essential to increase their variability and adaptability, which can help mitigate user fatigue and overcome implementation barriers. Additionally, addressing the interference caused by abnormal signals is critical for improving the accuracy of EEG data. Incorporating a range of signal types, including electromyographic and ocular activity, is vital for obtaining a comprehensive understanding of users’ physical and emotional states, thus enabling a more personalized interaction approach. Second, as research into BCI applications is still in its early stages, establishing a comprehensive database with standardized parameters is essential to facilitate training and application among researchers. Finally, the efficacy of BCI applications is heavily reliant on advanced deep learning models, underscoring the need for progress in training methodologies for EEG data to further enhance BCI performance. Future research should concentrate on developing multimodal hybrid closed-loop BCI models aimed at improving cognitive and executive functions related to motor tasks, promoting brain plasticity, and addressing the diverse rehabilitation needs of various patient populations. As advancements in artificial intelligence continue to unfold, we can anticipate the development of increasingly sophisticated BCI systems that not only transform rehabilitation practices but also deepen our understanding of brain functions, thereby expanding their applications across healthcare, entertainment, and educational sectors.
